# Supplementary material for: Innate immune activation by checkpoint inhibition in human patient-derived lung cancer tissues
Source: eLife. 2021 Aug 18;10:e69578. doi: 10.7554/eLife.69578 (PMC8476122; doi:10.7554/eLife.69578)
Supplement: Supplementary file 1. — Patient demographics and clinical characteristics. [file elife-69578-supp1.docx]

**Supplementary file 1. Table S1. Patient demographics and clinical characteristics.**

| **Patient #** | **UK131** | **UK2035** |
| --- | --- | --- |
| **Tumor Type** | Moderately differentiated squamous cell carcinoma | Metastatic large cell neuroendocrine carcinoma |
| **TNM Staging (pT)** | T1c | – |
| **TNM Staging (pN)** | N0 | – |
| **TNM Staging (pM)** | – | – |
| **Tumor Grade** | 2 | – |
| **FEV1/FVC ratio** | 74 | 58 |
| **Gender** | F | M |
| **Age** | 54 | 55 |
| **Ethnicity** | white | white |
| **Comorbidity** | COPD | COPD |
| **smoking history (pack years)** | 35 | – |
