# Supplementary material for: Innate immune activation by checkpoint inhibition in human patient-derived lung cancer tissues
Source: eLife. 2021 Aug 18;10:e69578. doi: 10.7554/eLife.69578 (PMC8476122; doi:10.7554/eLife.69578)
Supplement: Supplementary file 2. — Freshly resected CA lung tissue ofUK131 was FFPE-processed, sectioned as 4 μm slices, stained for KRT5 (squamous cellcarcinoma marker)/HIF1α in A, PD-1/CD8 in B, and PD-L1/CD206 in C, and analyzed byconfocal microscopy as described in Materials and methods. [file elife-69578-supp2.pdf]

Supplementary file 2

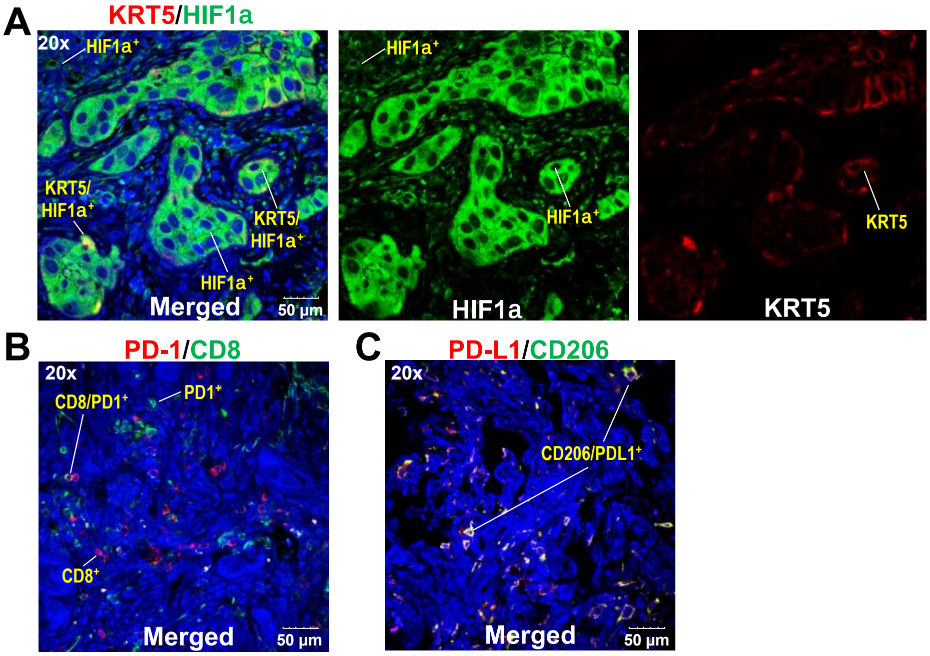

Fig. S1. Primary NSCLC tissue of UK131 patient stains positive for squamous cell carcinoma marker, PD-1 and PD-L1. Freshly resected CA lung tissue of UK131 was FFPE-processed, sectioned as 4 µm slices, stained for KRT5 (squamous cell carcinoma marker)/HIF1α in **A,** PD-1/CD8 in **B**, and PD-L1/CD206 in **C**, and analyzed by confocal microscopy as described in the Experimental.
