# Supplementary material for: Innate immune activation by checkpoint inhibition in human patient-derived lung cancer tissues
Source: eLife. 2021 Aug 18;10:e69578. doi: 10.7554/eLife.69578 (PMC8476122; doi:10.7554/eLife.69578)
Supplement: Supplementary file 3. — Freshly resected CA lung tissue of UK2035was FFPE-processed, sectioned as 4 μm slices, stained for CgA/NCAM1 (neuroendocrinetumor markers) in A and PD-1/PD-L1/CD8 in B, and analyzed by confocal microscopy, asdescribed in the Materials and Methods. [file elife-69578-supp3.pdf]

Supplementary file 3

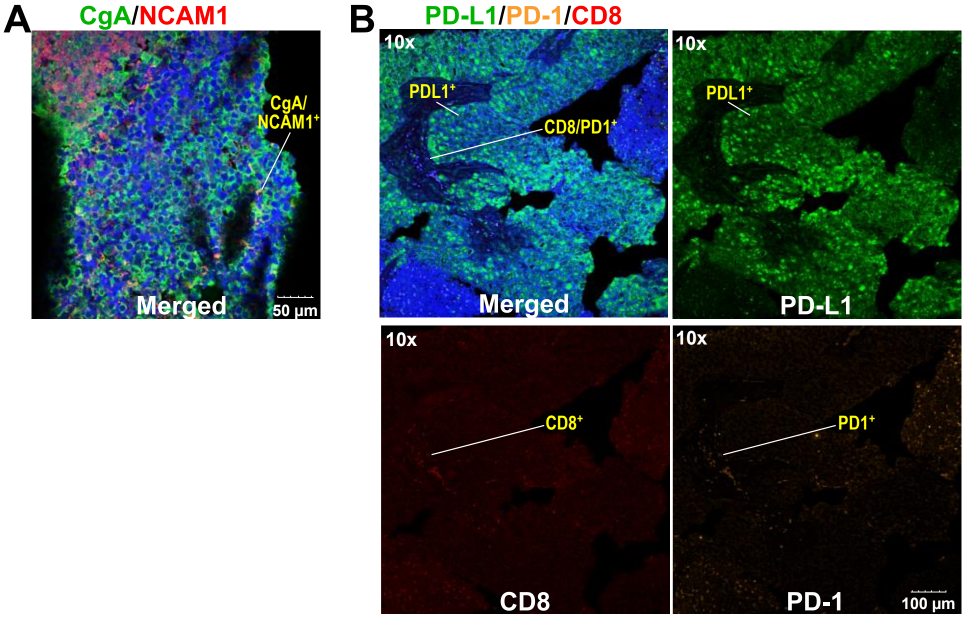

Fig. S2. Brain-metastasized NSCLC tissue of UK2035 patient stains positive for neuroendocrine markers, PD-1 and PD-L1. Freshly resected CA lung tissue of UK2035 was FFPE-processed, sectioned as 4 µm slices, stained for CgA/NCAM1 (neuroendocrine tumor markers) in **A** and PD-1/PD-L1/CD8 in **B**, and analyzed by confocal microscopy, as described in the Experimental.
