# Supplementary material for: Innate immune activation by checkpoint inhibition in human patient-derived lung cancer tissues
Source: eLife. 2021 Aug 18;10:e69578. doi: 10.7554/eLife.69578 (PMC8476122; doi:10.7554/eLife.69578)
Supplement: Supplementary file 4. — Information on primary antibodies used forimmunofluorescent staining. [file elife-69578-supp4.docx]

**Supplementary file 4. Table S2. Information on primary antibodies used for immunofluorescent staining.**

| **Antibody** | **Clone** | **Species** | **Source** | **Product #** | **Dilution** |
| --- | --- | --- | --- | --- | --- |
| PD-L1 | E1L3N | Rabbit | Cell Signaling | 13684 | 1:200 |
| PD-1 | EH33 | Mouse | Cell Signaling | 43248 | 1:200 |
| Cytokeratin 5 | 1A1C5 | Mouse | Proteintech | 66727-1-Ig | 1:10000 |
| CD8α | C8/144B | Mouse | Cell Signaling | 70306 | 1:200 |
| CD8α (DSP) | OTI3H6 | Mouse | Origene | CF802079 | 1:200 |
| CD68 (DSP) | KP1 | Mouse | SCBT | sc-20060 | 1:400 |
| HIF1a |  | Rabbit | Proteintech | 20960-1-AP | 1:50 |
| CD206 | D-1 | Mouse | Santa Cruz | sc-376108 | 1:100 |
| Chromogranin A | 3H11C11 | Mouse | Proteintech | 60135-1-Ig | 1:5000 |
| NCAM1/CD56 |  | Rabbit | Proteintech | 14255-1-AP | 1:2000 |
| PCNA | PC10 | Mouse | Cell Signaling | 2586 | 1:4000 |
| Pancytokeratin (DSP) | AE1/AE3 | Mouse | Invitrogen | 53-9003-82 | 1:500 |
| RIP | D94C12 | Rabbit | Cell Signaling | 3493 | 1:100 |
| Caspase 3 |  | Rabbit | Proteintech | 19677-1-AP | 1:200 |
